# Role of human milk banks amid COVID 19: perspective from a milk bank in India

**DOI:** 10.1186/s13006-020-00346-0

**Published:** 2020-12-02

**Authors:** Maheshwar Bhasin, Sushma Nangia, Srishti Goel

**Affiliations:** 1 Vatsalya: Maatri Amrit Kosh, National Comprehensive Lactation Management Centre, Lady Hardinge Medical College and Associated Hospitals, New Delhi, India; 2 Department of Neonatology, Lady Hardinge Medical College and Associated Hospitals, New Delhi, India

**Keywords:** COVID-19, SARS-CoV-2, Breastfeeding, Expressed breast milk, Human milk banks, Donor milk

## Abstract

The COVID-19 pandemic has had a significant impact on the operation of donor human milk banks in various countries such as China, Italy and India. It is understandable that this impact on operations of donor human milk might hamper the capability of these milk banks to provide sufficient pasteurized donor milk to neonates who need it. Contrary to developed world, predominant donors in developing nations are mothers of hospitalised neonates who have a relatively long period of hospital stay. This longer maternal hospital stay enhances the feasibility of milk donation by providing mothers with access to breast pumps to express their milk. Any excess milk a mother expresses which is above the needs of their own infant can be voluntarily donated. This physical proximity of milk banks to donors may help continuation of human milk donation in developing nations during the pandemic. Nevertheless, protocols need to be implemented to i) ensure the microbiological quality of the milk collected and ii) consider steps to mitigate potential consequences related to the possibility of the donor being an asymptomatic carrier of COVID-19. We present the procedural modifications implemented at the Comprehensive Lactation Management Centre at Lady Hardinge Medical College in India to promote breastfeeding and human milk donation during the pandemic which comply with International and National guidelines. This commentary provides a perspective from a milk bank in India which might differ from the perspective of the international donor human milk banking societies.

## Background

The novelty of the causative agent for COVID-19, the SARS-CoV-2 virus means that various aspects of the disease including its epidemiology, pathophysiology, clinical manifestations and management remain largely unknown. Clinical manifestations reported in COVID-19 patients include the full spectrum from asymptomatic carriers, to mild pneumonia-like symptoms, to severe respiratory distress or having a fatal outcome [1].

Pregnant women are immune suppressed and were reported to be more susceptible to respiratory infections including SARS-CoV and MERS-CoV [2, 3]. Adverse effects on pregnancy and perinatal outcomes such as sudden miscarriage, preterm labour, and intrauterine growth retardation have been well established in mothers with SARS-CoV [2].

Benefits of breastfeeding and human milk feeding for neonates have been well documented. The World Health Organization recommends observing necessary precautions for IPC (Infection, Prevention and Control) and continuing breastfeeding when a mother has, or is suspected of, COVID-19 infection [4]. Table 1 summarises the breastfeeding, expressed breast milk and donor milk guidelines from various organisations during the COVID-19 pandemic.

**Table 1:** Summary of clinical and milk banking recommendations regarding breastfeeding, breast milk, and donor human milk banking by various international and national organisations during the COVID-19 pandemic

Guidelines	Date published	Recommendations about breastfeeding/breast milk/ donor milk	Reference number
**International guidelines**			
UNICEF	–	Breastfeeding with necessary precautions; expressed breast milk if mother is too ill	[5]
World Health Organization	13 March 2020	Breastfeeding with necessary precautions	[4]
**National guidelines**			
China	3 February 2020	Isolate the mother and provide expressed breast milk	[6]
Switzerland	3 March 2020	Isolation of mother and no direct breastfeeding during 14 days of isolation	[7]
Italy	3 April 2020	Direct breastfeeding for asymptomatic and pauci-symptomatic mothers, expressed breast milk if mother is too sick.	[8]
Australia	29 March 2020	Breastfeeding with necessary precautions	[9]
India	1 April 2020	Breastfeeding with necessary precautions; expressed breast milk if isolation of mother is possible	[10]
United States of America	4 April 2020	Breastfeeding with necessary precautions, expressed breast milk	[11, 12]
	2 April 2020	Isolate the mother and provide expressed breast milk	[13]
Canada	11 April 2020	Breastfeeding with necessary precautions	[14]
**Milk banking guidelines**			
EMBA	25 February 2020	Rigorous donor screening. Safe to use breast milk. Donation suspended for symptomatic mothers	[15]
HMBANA	4 April 2020	Rigorous donor screening. Safe to use breast milk. Heat inactivation of virus and pasteurization efficacy	[16]

Davanzo [17], Chen [6] and Favre [7] from Italy, China, and Switzerland have expressed concern regarding breastfeeding amidst the COVID-19 pandemic. Whereas, Chinese guidelines are highly conservative, promoting separation of baby and mother with the use of Pasteurized Donor Human Milk (PDHM); some guidelines recommend use of expressed breast milk if feasible but no direct breastfeeding. Most others now recommend direct breastfeeding ensuring hand hygiene and use of face mask while nursing. Recommendations are to maintain physical separation at other times (maintain a distance of 2 m between mother and baby) in view of a concern related to the risk of SARS-CoV-2 transmission. A perspective dated 20 May 2020 by Boscia clearly reiterates direct breastfeeding with respiratory and hand hygiene by the mother [18].

The WHO scientific brief based on a systematic review including data prior to 15 May 2020, comprising of 46 mother baby dyads acknowledged lack of sufficient data but a low risk of SARS-CoV-2 transmission through breast milk culminating in the recommendation to initiate and continue direct breastfeeding for infants born to COVID-19 suspect/confirmed mothers [19]. The presence of viral RNA in breast milk has been reported by Wu et al. [20], Groß et al. [21], Kritsmen et al. [22] and Chambers et al. [23] whereas multiple other studies did not detect the presence of viral RNA in breast milk [24–29]. However, Chambers et al. further report the absence of viable RNA on cell culture and complete inactivation of viral particles through Holder pasteurization (a common process in human milk banks) [23]. These observations were subsequently fully supported by Unger et al. [30].

Breast milk possesses numerous bioactive components including immunoglobulins, lactoferrin, lysozymes, oligosaccharides and microRNA. The presence of protective antibodies against SARS-CoV and specifically against receptor-binding domain of spike protein of SARS-CoV-2 has been reported in various studies [31–34] although the antibody expression pattern in breast milk remains unclear [24]. Although case reports of infants testing positive for SARS-CoV-2 at 36 h, 15 days, 17 days, 55 days and 3 months of life [35–39] raise concerns of horizontal transmission, the existing evidence does not support vertical transmission of SARS-CoV-2 or infection through breast milk and the recent commentary by Gribble et al. [40], promotes and supports the recommendations of the WHO for continuing breastfeeding.

COVID-19 suspicion or positivity in a mother, her isolation to a COVID ward and surrounding uncertainties during parturition have unfortunately resulted in mothers being separated from their infants. Under such circumstances, direct breastfeeding and expressing breast milk may not be possible, donor human milk has been considered as the best alternative. An initial guideline also suggests the consideration of donor human milk [41]. However, concerns over feasibility of such a strategy and capability of donor human milk banks to sustain all such babies have been shared by various experts [42–44].

In the recent commentary regarding an international perspective on donor human milk banking during COVID-19 pandemic in the *Journal of Human Lactation*, Marinelli explores the challenges faced by the international donor human milk banking community [42]. It highlights the risk involved and shortage of supply of raw milk to donor human milk banks. However, this commentary does not reflect upon the possible role of human milk banks in promoting breastfeeding and providing donor milk as gap support to isolated infants in NICU and PICU. The commentary also does not reflect upon precautions to be observed by human milk bank personnel and advantages of hospital based human milk bank setups. Finally, the reflections are based on human milk bank setups in developed nations.

Moro et al. from Italy shares the negative impact of COVID-19 on donor human milk bank systems [44]. In a news article shared by regional centre in Jaipur, India, a decline of 75% in donor human milk collected was reported between 23 March and 8 June 2020. Informal conversations between centres across the nation highlight similar observations. It is to be evaluated carefully whether the root causes of decline can be attributed to nationwide lockdown measures, stigma and uncertainty associated with COVID-19 or if the decline is relative to a decreased number and hospital stay of patients. This commentary aims to explore the role of human milk banks in promoting breastfeeding and providing options for the supply of milk for neonates during the COVID-19 pandemic in low- and middle-income countries (LMICs) like India by sharing the experiences and challenges from Vatsalya Maatri Amrit Kosh at Lady Hardinge Medical College, New Delhi, India.

### Structure of milk banks in India

**Fig. 1:**
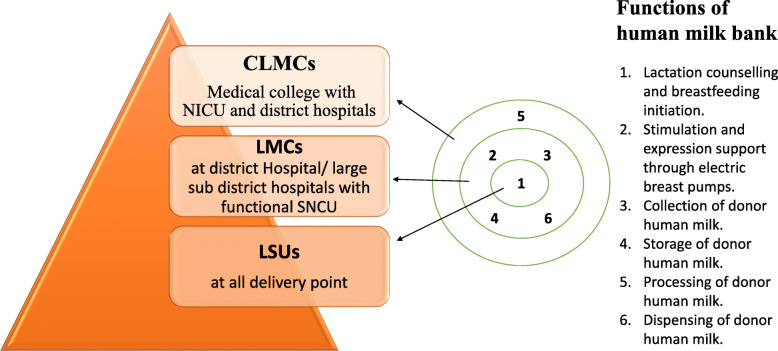
Levels of facility-based Lactation Management Centres in India. Functions of a human milk bank include lactation support and consultation, expression by electric pumps, collection, storage and processing of human milk and dispensing to NICUs. According to the Indian guidelines for CLMCs, electric breast pumps are provided in LMCs and CLMCs and pasteurization units (Milk Processing) is limited to CLMCs. Milk collected at LMCs is to be transported to CLMCs from where processed milk is sent back to LMCs. (Adapted from the National Guidelines on Lactation Management Centres in Public Health Facilities [45])

In India, human milk banks are established as Lactation Management Centres (LMC) at three levels such as, Lactation Support Units (LSU) at delivery points, Lactation Management Units (LMU) at district levels with functional Special Newborn Care Units (SNCU) and Comprehensive Lactation Management Centres (CLMC) at a tertiary level (Fig. 1). India now has nearly 80 milk banks, operational as per the National Guidelines on Lactation Management Centres in Public Health Facilities [45].

The donation of milk is a non-incentivised process and remains a completely voluntary activity. The guidelines condemn the usage of donated human milk for commercial purposes. The protocol for screening of donors is stringent and concurs with the donation policies of other human milk banking associations [15]. The processing of milk in the milk banks including storage, thawing, pasteurization, testing and storage till disbursement, all follow good manufacturing practices. Quality assurance of the process accompanied by a detailed food safety approach elaborated as Hazard Analysis and Critical Control Points are extensively detailed in the guidelines and adhered to in the milk banking process [45].

### Scenarios during COVID-19 pandemic

#### Expression

The predominant way of expressing milk for an infant who cannot breastfeed directly in LMIC is hand expression. In accordance with the interim guidelines for breastfeeding during COVID-19, maintaining proper hand hygiene is strictly enforced [46]. The expression of milk by pumping is usually supervised by a lactation counsellor during daytime hours. However, due to resource limitation mothers usually need to hand express their milk during the night.

At our centre, a CLMC, the milk expressed using breast pumps under lactation counsellor’s supervision is usually sent back to the NICU to feed mother’s own milk to her own baby. The electric breast pumps are currently stationed in CLMC due to space and cost constraints. The mother comes to CLMC to express her milk through pumping. A mother’s milk is donated only if it is in excess of her own infant’s need and the mother is willing to donate. This presents a unique opportunity for human milk banks to play a quintessential role in ensuring the adherence to recommendations of hand hygiene and measures under IPC while expressing the milk. This also relieves the NICU staff of additional burden of expression and taking care of pumps and safeguards that the benefits of mother’s own milk are passed onto the neonate.

The general hygienic practices implemented at LMUs and CLMCs ensure cleanliness and prevent cross contamination. In addition, during this pandemic a few experts emphasised practices for mothers to follow at our centre including:

1. Before entering, a mother is verbally screened for symptoms of flu-like illness by a health care professional. If the mother is symptomatic, she is referred to the COVID-19 screening facility at the hospital and is only allowed to express if reported negative for COVID-19.
2. Adherence of mothers and milk bank staff to social distancing during expression of milk and while practicing hand hygiene.
3. A limited number of mothers and health care professionals are allowed in the expression room.
4. Milk Bank staff who can work from home have been relieved and only called on duty, if necessary. Our centre is fully functional at its maximum capacity with the minimum number of necessary staff.
5. Face masks are worn by health care professionals and mothers at all times.
6. Lactation counsellors are provided with personal protective equipment (PPE) for assisting a Covid-19 suspect or positive mother during pumping to avoid unnecessary contact with surface. (A dedicated lactation counsellor in PPE helps and ensures mothers express their breast milk hygienically and use the correct technique during their hospital stay. If a mother is sick requiring hospitalisation then although she cannot breastfeed directly, her expressed milk is used but if she is on respiratory support then she is unable to breastfeed directly or express her milk and the baby receives PDHM or formula milk depending on gestation and sickness level of the baby. Contraindications are by far and large the same as those for COVID-19 negative mother.)
7. Autoclaved closed containers are supplied to the milk bank from the NICU and mothers carry their expressed milk in those closed containers to the NICU.

#### Donation

Reportedly, amidst the ongoing COVID-19 pandemic, the international donor human milk banking community is impacted by COVID-19preventative measures [42–44]. The practices followed at donor human milk banks ensure the safety of donor human milk. The negative impact shared by Moro et al. [44] and in India may be due to poor access to blood collection of new donors as well as issues with the collection and delivery of donated milk from home.

In India and many other LMICs, predominant donors to human milk banks at tertiary care hospitals are mothers who have given birth to infants requiring NICU admissions [47]. These donors express the milk in human milk banks, and during this ongoing pandemic, have continued to stay at the hospital. This is contrary to donor human milk banks in other countries where mothers tend to express their milk at home and transport it to the milk banks. The presence of mothers with preterm infants in the hospitals provides a unique opportunity for human milk banks such as ours to act as a reservoir of donor human milk.

As a precautionary step, questions about possible travel history, history of contact with a COVID-19 positive person, residence in a containment area or belonging to a hotspot zone along with presence of flu-like illness have been added to the existing donor registration form. These are similar to the questions asked for non-donor mothers who visit the centre to express with breast pumps.

#### Pasteurization

The process of handling milk in a milk bank requires good manufacturing practices and the use of PPE to protect the personnel and donated human milk from unnecessary exposure. Donor screening and microbiological profiling of raw donor human milk and pasteurization ensures the safety of the PDHM. As elaborated by the European Milk Banking Association (EMBA) in its statement, the process of pasteurization could potentially help destroy SARS-CoV-2, if present [15]. The statement is supported by recent studies demonstrating complete elimination of viral load after exposure at 56 °C for 30 mins [23, 30, 48].

#### Hygiene helpers

Hygiene helpers are responsible for cleaning plasticware in CLMCs. In view of the fact that mothers visiting the facility could potentially be asymptomatic carriers of SARS-CoV-2, appropriate protective gear like face shields, N-95 masks, gowns and gloves are donned by the hygiene helpers while handling the plasticware utilized for mothers during milk expression.

Human milk banks should maintain impeccable hygiene standards at all times. With the well debated issue surrounding the handling of human milk containers as being potential sources of SARS-CoV-2 transmission, Marinelli et al. suggested using “high level of disinfection” bleach to clean plasticwares in milk banks [42]. Alternatively, HMBANA suggested the bottle transfer technique [49]. In an article by Moro et al. [44], the author shares the notion of single use disposable bottles in Italy and suggests exploring the potential of cleaning outside surface of the bottles with silver ions as suggested by Peila et al. [50]. In resource limited environment, implementation of many of these suggestions including single use bottle might not be economically feasible.

What is interesting to note is that the temperature stability study reports complete inactivation of SARS-CoV-2 virus after 5 min of exposure at 70 degrees Celsius [48]. The process of cleaning the lactasets (i.e. breast shields, membranes, valves, connectors, and tubes) and bottles in settings similar to ours includes dipping them in soap solution before rinsing under running water. Thereafter, the plastic wares are kept in boiling water (100 degrees Celsius) for 20 min and dried using a hot air oven. The relevance of this cleaning process in context with SARS-CoV-2 is still to be explored.

## Conclusion

The ongoing pandemic has impacted the functioning of human milk banks throughout the world. Attempts should be made to minimise the impact on the health and well-being of the vulnerable infants. Breast milk has notable benefits for the neonate including the potential for transfer of bioactive agents such as anti-infective antibodies along with other established long-term benefits. The core belief of human milk banking across the globe is to promote and strengthen breastfeeding.

Human milk banks in developing nations have a pivotal role during these unpropitious circumstances to facilitate the use of breast milk and ensure provision of donor milk as gap support to the most vulnerable preterm infants (< 1500 g and < 32 weeks gestation) if mother’s own milk is not available.

## Data Availability

Data sharing is not applicable to this article as no datasets were generated or analysed during the current study.

## Abbreviations

CLMC: Comprehensive Lactation Management Centre
COVID 19: Coronavirus Disease 2019
EMBA: European Milk Banking Association
LMU: Lactation Management Unit
LSU: Lactation Support Unit
MERS-CoV: Middle Eastern Respiratory Syndrome Corona Virus
PDHM: Pasteurized Donor Human Milk
SARS-CoV: Severe acute respiratory syndrome Corona Virus
SDH: Sub District Hospitals
SNCU: Special Newborn Care Units
